# Molecular evidence of *Rickettsia raoultii*, “*Candidatus* Rickettsia barbariae” and a novel *Babesia* genotype in marbled polecats (*Vormela peregusna*) at the China-Kazakhstan border

**DOI:** 10.1186/s13071-018-3033-z

**Published:** 2018-08-04

**Authors:** Xiafei Liu, Meihua Yang, Guangyuan Liu, Shanshan Zhao, Wumei Yuan, Ronghai Xiao, Wurelihazi Hazihan, Sándor Hornok, Yuanzhi Wang

**Affiliations:** 1 School of Medicine, Shihezi University, Shihezi, Xinjiang Uygur Autonomous Region 832002 People’s Republic of China; 2 School of Agriculture, Shihezi University, Shihezi, Xinjiang Uygur Autonomous Region 832000 People’s Republic of China; 3 State Key Laboratory of Veterinary Etiological Biology, Key Laboratory of Veterinary Parasitology of Gansu Province, Lanzhou Veterinary Research Institute, Chinese Academy of Agricultural Science, Xujiaping 1#, Lanzhou, Gansu 730046 People’s Republic of China; 4 Inspection and Comprehensive Technology Center of Ruili Entry-Exit Inspection and Quarantine Bureau, No.75, Ruihong Road, Ruili, 678600 Yunnan People’s Republic of China; 5 School of Animal Science and Technology, Shihezi University, Shihezi, Xinjiang Uygur Autonomous Region 832000 People’s Republic of China; 6 Department of Parasitology and Zoology, University of Veterinary Medicine, Budapest, Hungary

**Keywords:** *Babesia*, “*Candidatus* Rickettsia barbariae”, China-Kazakhstan border, *Haemaphysalis erinacei*, Marbled polecat, *Rickettsia raoultii*

## Abstract

In this study, two road-killed marbled polecats (*Vormela peregusna*) were molecularly analysed for tick-borne pathogens. *Rickettsia raoultii*, “*Candidatus* Rickettsia barbariae” and a novel *Babesia* genotype have been identified, for the first time in marbled polecat. DNA of this *Babesia* sp. genotype was also present in four out of 15 *Haemaphysalis erinacei* ticks collected from the *Babesia* PCR-positive marbled polecat. Results of this study suggest that marbled polecats may serve as reservoirs for these bacteria and protozoans.

**Electronic supplementary material:**

The online version of this article (10.1186/s13071-018-3033-z) contains supplementary material, which is available to authorized users.

## Letter to the Editor

The marbled polecat (*Vormela peregusna*) is a small carnivorous mammal (Carnivora: Mustelidae) with a broad geographical range, extending from southeast Europe, through southwest and central Asia, to Mongolia and northern China. This species is the only member of the genus and has been listed as globally vulnerable, due to substantially declining populations [1]. Apart from the loss of steppe habitats and desertification, infection with various pathogens may contribute to its decreasing numbers. At the same time, data are very limited on the epidemiological role of the marbled polecat as a reservoir of pathogens with veterinary or medical significance [2, 3]. Therefore, molecular investigations for pathogens in this endangered species have multifold importance.

In May and June 2014, two road-killed, female marbled polecats, were found around wetlands of Ebinur Lake (189 m above sea level; coordinates: 82°48'51"E, 45°04'22"N) in northwestern China, in the border region near Kazakhstan. Previously, 21 *Haemaphysalis erinacei* ticks collected from the two animals (15 from polecat #1 and six from polecat #2) were molecularly characterised at 16S mitochondrial gene region. Two of 15 ticks (13.33%) collected from marbled polecat #1 were infected with *Rickettsia raoultii* [4]. The two animals were brought to the Xinjiang Uygur Autonomous Region Wildlife Management Office and then sent to the Laboratory of High Incidence of Local and Ethnic Diseases in Xinjiang for necropsy analyses. DNA extractions from the liver and spleen were carried out using the TIANamp Genomic DNA Kit (TIANGEN, Beijing, China). The presence of DNA from tick-borne pathogens was investigated by PCR amplification and sequencing of parts of the following genes: the 17-kDa surface antigen gene (*17 kDa* gene) of *Rickettsia* spp., the *5S-23S* rRNA gene of *Borrelia* spp.*,* the *16S* rRNA gene of Anaplasmataceae, and the *18S* rRNA gene of *Babesia* spp., as described previously [5–7]. All samples were negative for *Borrelia* spp. and Anaplasmataceae. To confirm the results and to compare additional genetic markers, further PCR and sequencing were performed, to detect the outer membrane protein A (*ompA*) and cell surface antigen 1 (*sca*1) genes of *Rickettsia* spp. [5] and the c*ytochrome b* (*cytb*) gene of *Babesia* spp. [8]. All PCRs were performed including double distilled water (ddH_2_O) as a negative control. Sequences were compared with GenBank data using the nucleotide BLAST program (http://www.ncbi.nlm.nih.gov/BLAST/). All obtained sequences were deposited in the GenBank database [*17-kDa*: MG674917 (*R. raoultii*) and MG674918 (“*Candidatus* Rickettsia barbariae”); *ompA*: MG662380 (*R. raoultii*) and MG662381 (“*Candidatus* Rickettsia barbariae”); *sca*1: MG662382 (*R. raoultii*) and MG662383 (“*Candidatus* Rickettsia barbariae”); *18S* rRNA: MG799848 and MG813565; *cytb:* MG832590 and MG832591 (*Babesia* sp. from marbled polecat and *H. erinacei*, respectively)].

**Fig. 1:**
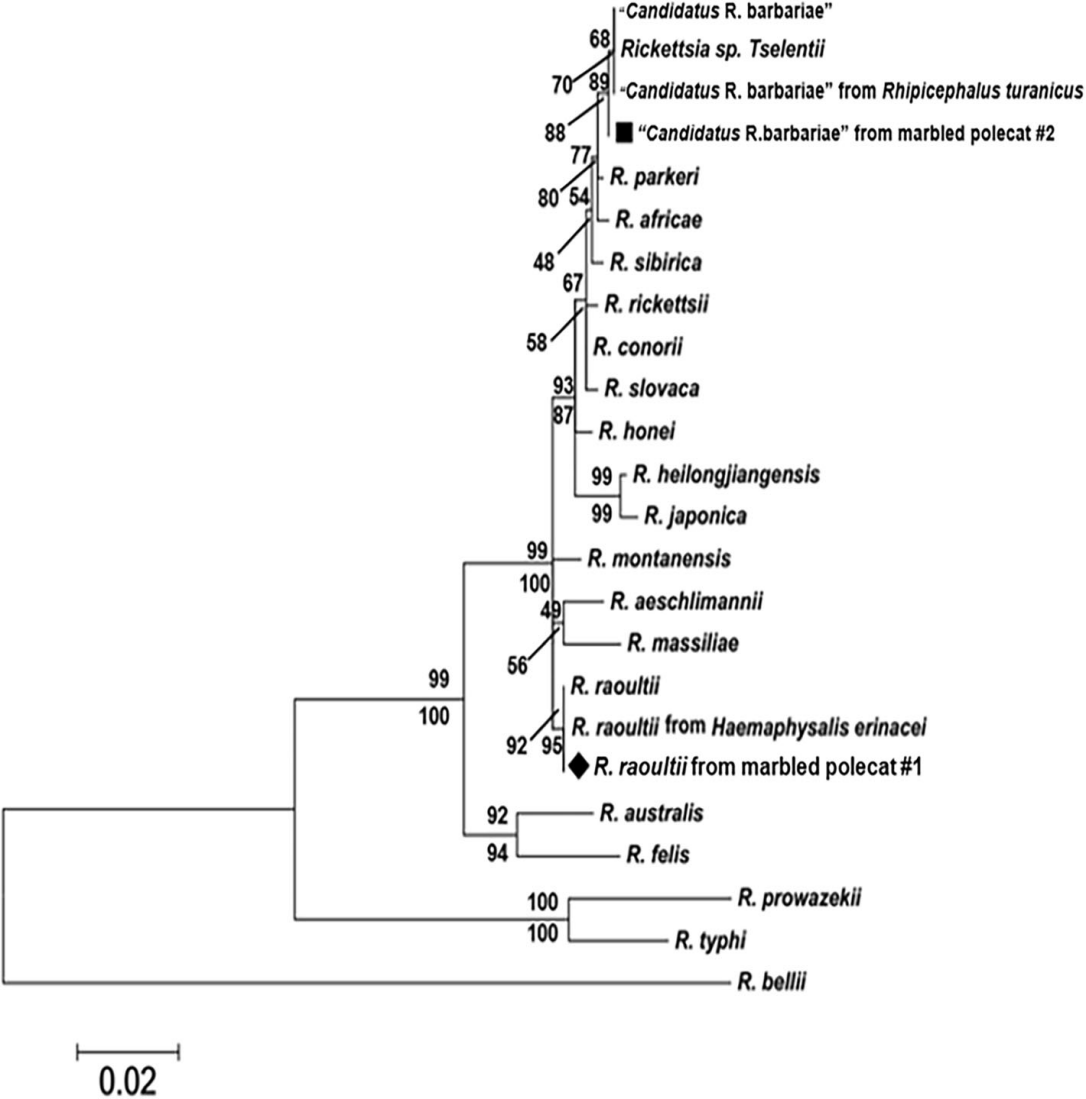
Phylogenetic tree of the *17-kDa* (393 bp) - *ompA* (443 bp) - *sca1* (553 bp) concatenated sequences for “*Candidatus* Rickettsia barbariae” (indicated by a square) and *R. raoultii* (indicated by a diamond) from the marbled polecat obtained in this study, and sequences for *Rickettsia* species retrieved from the GenBank database. The tree was constructed using the neighbour-joining method (NJ; 1000 bootstrap replicates) and maximum-likelihood (ML; 1000 bootstrap replicates) analyses using MEGA6. The scale-bar represents the inferred substitutions per nucleotide site. The relative support for clades in the tree was produced from the NJ and ML analyses

However, the liver and spleen samples of both marbled polecats were PCR-positive for rickettsiae. In animal #1, the *ompA* and *sca*1 sequences showed 100% identities with *R. raoultii* strain Khabarovsk^T^ (GenBank: CP010969). This result is in line with previous findings, i.e. two out of the fifteen ticks (i.e. 13.33%) collected from animal #1 harboured *R. raoultii*. The phylogenetic analysis showed that the genetic pattern was the same between *R. raoultii* from a marbled polecat #1 and *R. raoultii* previously found in *H. erinacei* ticks collected from marbled polecat #1 (Fig. 1) [4]. In animal #2, “*Candidatus* Rickettsia barbariae” was identified, with 99.8% (552/553 bp) and 100% (443/443 bp) similarities to sequences available in GenBank [from *Vermipsylla alakurt* in China: KT284718 (*sca*1) and KU645284 (*ompA*)]. Results of sequence alignments were confirmed by phylogenetic analyses, placing rickettsiae detected here into the cluster of either *R. raoultii* or “*Candidatus* Rickettsia barbariae” (Fig. 1 and Additional file 1: Table S1).

Both *Rickettsia* spp. were detected for the first time in marbled polecats. Spotted fever group rickettsiae (Proteobacteria: Rickettsiales) are obligatory intracellular, Gram-negative bacteria, which can cause zoonotic disease after transmission by a blood-sucking arthropod [9]. *Rickettsia raoultii* is one of the causative agents of human rickettsioses (SENLAT/TIBOLA/DEBONEL [10]). Until recently, *R. raoultii* has not been reported from mammals other than humans [11], but during the past years, this species was detected in Mongolian gazelle [12] and dogs [13]. The most important tick vectors of *R. raoultii* appear to be *Dermacentor* spp., with transstadial and transovarial maintenance of this agent [14]. Accordingly, a previous study revealed *R. raoultii* as the predominant species of *Rickettsia* found in *D. nuttalli* from Mongolian regions and in *D. silvarum* ticks in the border region between China and Russia [15, 16]. Therefore, simultaneous detection of *R. raoultii* in a marbled polecat (as shown here) and in *H. erinacei* removed from the same animal justify further studies on the vector role of this tick species, which is outside the typical vector range of *R. raoultii*.

“*Candidatus* Rickettsia barbariae” has been detected and described from ticks of domestic and wild animals [17–20] and humans [21] in the Mediterranean region. Our previous work also showed the presence of DNA of “*Candidatus* Rickettsia barbariae” in the tick *Rhipicephalus turanicus* [22] and the flea *Vermipsylla alakurt* [5] collected from sheep around the Taklamakan Desert in Xinjiang, northwestern China. To the best of our knowledge, “*Candidatus* Rickettsia barbariae” was not detected in any vertebrate hosts of the above ectoparasites, further increasing the significance of the present findings.

**Fig. 2:**
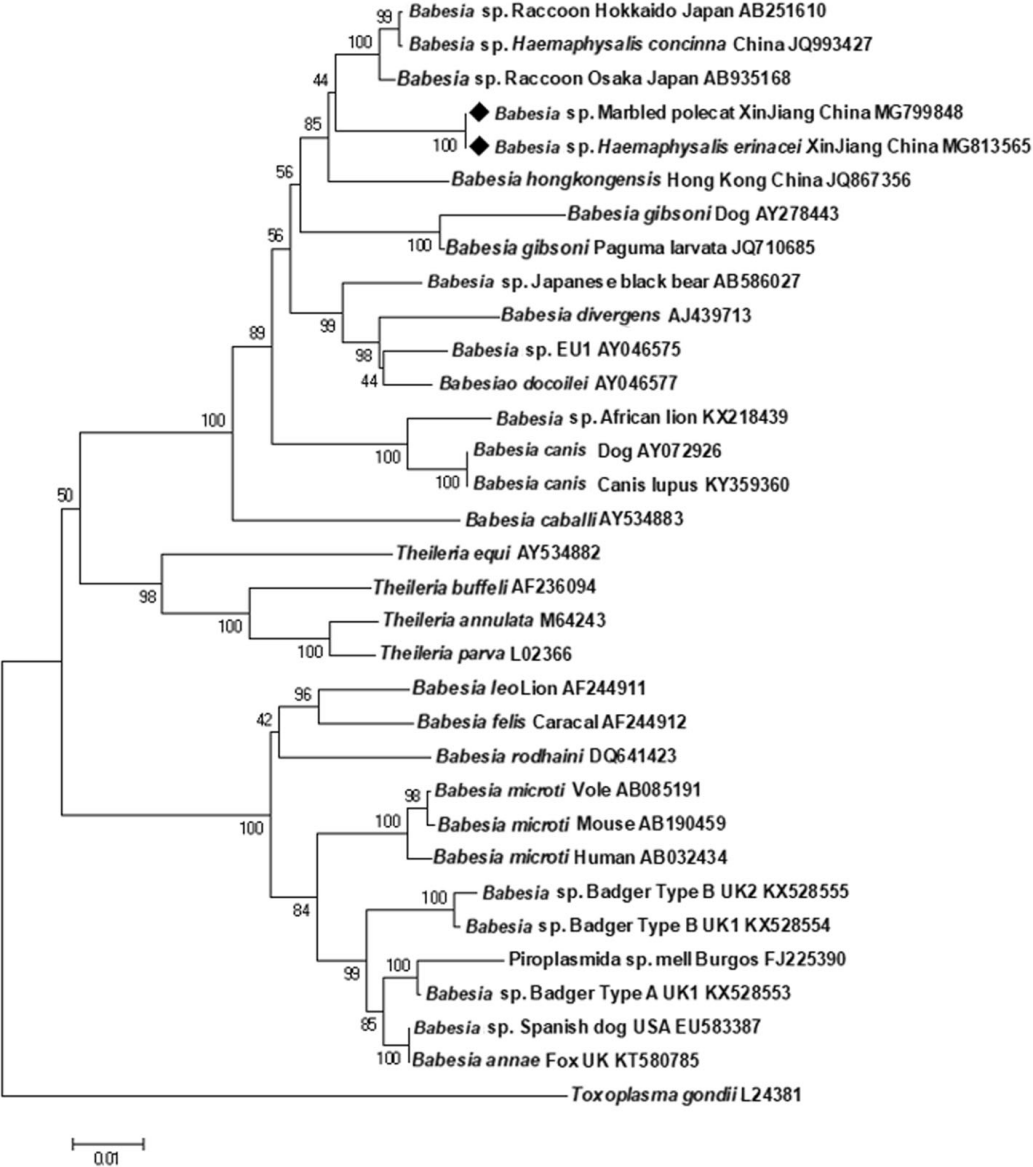
Phylogenetic comparison of 1601 bp *18S* rRNA gene sequence of *Babesia* sp. from the marbled polecat and *Babesia* sp. *Haemaphysalis erinacei* identified in the present study (diamonds) and relevant sequences from the GenBank database. The results are based on the neighbour-joining (NJ; 1000 bootstrap replicates) approximation of the standard likelihood ratio test score. The scale-bar indicates the base pair substitution rate

In addition to the above results, *Babesia* sp. DNA was detected both in four *H. erinacei* ticks and the organs of their parasitised host (marbled polecat #1). The *18S* rRNA gene sequences were 100% identical between the marbled polecat and its PCR-positive ticks. However, this *Babesia* genotype had only 97.12% (1517/1562 bp) *18S* rRNA gene similarity to the closest genotype in GenBank, detected in the blood of a racoon in Japan (GenBank: AB935168). The *cytb* gene sequence comparisons supported the uniqueness of this *Babesia* sp. from marbled polecat because the gene sequence showed 85.74% (433/505 bp) similarity to that of *Babesia gibsoni* (no corresponding *cytb* gene sequence was found for *Babesia* sp. from a racoon in GenBank). The phylogenetic analysis confirmed these results: this novel *Babesia* genotype clustered separately from the above racoon-associated piroplasm (GenBank: AB935168) and belonged to a phylogenetic group including *Babesia* spp. from Caniformia or tick species (i.e. *H. concinna* and from the present study *H. erinacei*) frequently infesting Caniformia (Fig. 2). The separation of this phylogenetic group from a cat-associated piroplasm (*B. hongkongensis*) was strongly (85%) supported (Fig. 2).

To the best of our knowledge, this is the first report on the presence of *Babesia* DNA in marbled polecat and *H. erinacei* ticks. *Babesia* spp. (Apicomplexa: Piroplasmida) are intraerythrocytic parasites, which have been reported from birds and mammals (including wild carnivores) worldwide [7, 23] and have ixodid ticks as their principal vectors [24]. The *18S* rRNA gene is the most widely used genetic marker for the identification of babesiae [24]. Based on *18S* rRNA gene sequence data and the topology of the phylogenetic tree (Fig. 2), the *Babesia* sp. detected here in the marbled polecat, and the attendant ticks was most closely related to a *Babesia* sp*.* from a racoon (GenBank: AB935168: reported from Osaka, Japan) and differed from all other piroplasmid species. Taking into account that the *18S* rRNA gene may have very few nucleotide substitutions between closely related species [e.g. only 0.2% difference delineating *B. divergens* (GenBank: FJ944825) and *B. capreoli* (GenBank: AY726009) [25]], this new *Babesia* genotype most likely represents a species different from the one detected in raccoon in Japan. Unfortunately, morphological characterisation of this novel *Babesia* genotype was not possible because samples were obtained from road-killed (i.e. not freshly dead) animals, and *Babesia* are known to undergo quick degradation and morphological changes post-mortem [26].

In conclusion, results of this study suggest that marbled polecats may serve as reservoirs for *R. raoultii*, “*Candidatus* Rickettsia barbariae” and a novel *Babesia* genotype. Further studies are needed to evaluate if rickettsemia in this host species is of sufficient magnitude and duration to infect ticks, which is a known prerequisite for effective transmission of other tick-borne rickettsiae [27].

## Additional file

Additional file 1: **Table S1.** Information for the sequences from the GenBank database used in Fig. 1. (DOCX 18 kb)

## Abbreviations

cytb: cytochrome b
DEBONEL: *Dermacentor*-borne necrosis, eschar and lymphadenopathy
HGA: human granulocytic anaplasmosis
IUCN: International Union for Conservation of Nature
ompA: outer membrane protein A
sca1: surface cell antigen 1
TIBOLA: tick-borne lymphadenopathy
