# Supplementary material for: Molecular evidence of Rickettsia raoultii, “Candidatus Rickettsia barbariae” and a novel Babesia genotype in marbled polecats (Vormela peregusna) at the China-Kazakhstan border
Source: Parasit Vectors. 2018 Aug 4;11:450. doi: 10.1186/s13071-018-3033-z (PMC6090811; doi:10.1186/s13071-018-3033-z)
Supplement: Supplementary file 1 — Table S1. Information for the sequences from the GenBank database used in Fig. 1. (DOCX 18 kb) [file 13071_2018_3033_MOESM1_ESM.docx]

**Additional file 1: Table S1.** Information for the sequences from the GenBank database used in Fig. 1

| *Rickettsia* spp. | *17-**kDa*  (accession No.) | *ompA*  (accession No.) | *sca1*  (accession No.) |
| --- | --- | --- | --- |
| *R. raoultii*  from *Vormela peregusna* 1# | MG674917 | MG662380 | MG662382 |
| *R. raoultii*  from Haemaphysalis erinacei | KR608784 | KR608786 | KR608788 |
| *R. raoultii* | CP019435 | CP019435 | CP019435 |
| “*Candidatus* Rickettsia barbariae” from *Vormela peregusna* 2# | MG674918 | MG662381 | MG662383 |
| “*Candidatus* Rickettsia barbariae” from *Veripsylla alakurt* | KT284715 | KU645284 | KT284718 |
| “*Candidatus* Rickettsia barbariae” | MF002507 | MF002506 | MF002505 |
| *Rickettsia* sp. Tselentii | GU353184 | EU194445 |  |
| *R**. parkeri* | CP003341 | CP003341 | CP003341 |
| *R.africae* | CP001612 | CP001612 | CP001612 |
| *R.sibirica* | MF002548 | U43807 | MF002544 |
| *R.rickettsii* | CP018914 | CP018914 | CP018914 |
| *R.conorii* | AE006914 | AE006914 | AE006914 |
| *R.slovaca* | CP003375 | CP003375 | CP003375 |
| *R.honei* | AF060704 | U43809 | AY355351 |
| *R.heilongjiangensis* | CP002912 | CP002912 | CP002912 |
| *R.japonica* | AP017602 | AP017602 | AP017602 |
| *R.montanensis* | CP003340 | CP003340 | CP003340 |
| *R.aeschlimannii* | DQ379977 | MF379308 | AY355353 |
| *R.massiliae* | CP003319 | CP003319 | CP003319 |
| *R.raoultii* | CP019435 | CP019435 | CP019435 |
| *R.australis* | CP003338 | CP003338 | CP003338 |
| *R.felis* | CP000053 | CP000053 | CP000053 |
| *R**.prowazekii* | CP014865 |  | CP014865 |
| *R.**typhi* | CP003398 |  | CP003398 |
| *R.bellii* | CP000087 |  | CP000087 |
